# CellAnn: a comprehensive, super-fast, and user-friendly single-cell annotation web server

**DOI:** 10.1093/bioinformatics/btad521

**Published:** 2023-08-23

**Authors:** Pin Lyu, Yijie Zhai, Taibo Li, Jiang Qian

**Affiliations:** Department of Ophthalmology, Johns Hopkins University School of Medicine, Baltimore, MD 21287, United States; Department of Ophthalmology, Johns Hopkins University School of Medicine, Baltimore, MD 21287, United States; Department of Biomedical Engineering, Johns Hopkins University School of Medicine, Baltimore, MD 21218, United States; Department of Ophthalmology, Johns Hopkins University School of Medicine, Baltimore, MD 21287, United States

## Abstract

**Motivation:**

Single-cell sequencing technology has become a routine in studying many biological problems. A core step of analyzing single-cell data is the assignment of cell clusters to specific cell types. Reference-based methods are proposed for predicting cell types for single-cell clusters. However, the scalability and lack of preprocessed reference datasets prevent them from being practical and easy to use.

**Results:**

Here, we introduce a reference-based cell annotation web server, CellAnn, which is super-fast and easy to use. CellAnn contains a comprehensive reference database with 204 human and 191 mouse single-cell datasets. These reference datasets cover 32 organs. Furthermore, we developed a cluster-to-cluster alignment method to transfer cell labels from the reference to the query datasets, which is superior to the existing methods with higher accuracy and higher scalability. Finally, CellAnn is an online tool that integrates all the procedures in cell annotation, including reference searching, transferring cell labels, visualizing results, and harmonizing cell annotation labels. Through the user-friendly interface, users can identify the best annotation by cross-validating with multiple reference datasets. We believe that CellAnn can greatly facilitate single-cell sequencing data analysis.

**Availability and implementation:**

The web server is available at www.cellann.io, and the source code is available at https://github.com/Pinlyu3/CellAnn_shinyapp.

## 1 Introduction

Single-cell RNA-sequencing (scRNA-seq) is a genomic method to detect gene expression levels at the single-cell level. Even though it was invented <10 years ago, it has been widely used to identify novel cell types and cell heterogeneity during development and disease (Saliba *et al.* 2014; Wen and Tang 2016; Baslan and Hicks 2017; Ofengeim *et al.* 2017; Rozenblatt-Rosen *et al.* 2017; Potter 2018). The common practice in scRNA-seq data analysis is first to cluster the cells based on the similarity of the gene expression profiles in each cell. The next step is cell annotation, assigning the specific cell types to the clusters. Even though cell annotation is essential to understand the biological properties of the cells, it is challenging because it requires domain knowledge in specific cells or tissues.

Two major approaches exist for cell annotation. One approach utilizes the known marker genes for specific cell types. Marker genes can be found in online databases such as CellMarker (Zhang *et al.* 2019b) and PanglaoDB (Franzén *et al.* 2019). Popular methods in this category include ScType (Ianevski *et al.* 2022), scSorter (Guo and Li 2021), CellAssign (Zhang *et al.* 2019a), and scCATCH (Shao *et al.* 2020). However, this approach is not always successful due to the limited knowledge of marker genes of some cell types. For example, some poorly studied cell types have very few or no marker genes. In addition, some known marker genes might not be specific to a cell type as expected. The second type of cell annotation approach is based on the reference datasets. Several methods have been developed for this purpose, including scClassify (Lin *et al.* 2020), Scibet (Li *et al.* 2020), singleCellNet (Tan and Cahan 2019), scMAGIC (Zhang *et al.* 2022), and singleR (Aran *et al.* 2019). This type of approach takes advantage of the published datasets that were carefully studied by domain experts. Instead of explicitly extracting the marker genes associated with each cluster, the cell types are characterized by the gene expression profiles defined by many variable genes. By comparing the expression profiles of query clusters and annotated reference datasets, the cell types can be “borrowed” from the reference datasets if the gene expression profiles of the query and reference are similar enough.

A good reference-based cell annotation system should have the following desirable features. First, we need a large set of preprocessed reference datasets, which makes it easy for the users to find the relevant reference datasets for their query dataset. Unfortunately, most available tools rely on the users to identify, download and process the reference dataset before the users can perform the analysis. Second, a good annotation system should not require sophisticated computational skills to run the task. However, most reference-based methods require users to install the tools on their computers. Some tools are only available in specific computational languages such as Python or R. Third, an ideal method should run fast even with large reference or query datasets, and it should not require a large computer memory. Some advanced methods, such as those employing deep learning approaches, have been developed and have a good performance [scBERT (Yang *et al.* 2022), scDeepSort (Shao *et al.* 2021), ACTINN (Ma and Pellegrini 2020), sigGCN (Wang *et al.* 2021), scIAE (Yin *et al.* 2022), scNym (Kimmel and Kelley, 2021), SuperCT (Xie *et al.* 2019), and EnClaSC (Chen *et al.* 2020)]. However, these methods are often slow and require large memories and computing resources, making them not suitable for an online tool. Furthermore, many single-cell datasets are generated from droplet-based platforms, which typically include cells on the scale of hundreds of thousands or larger (Macosko *et al.* 2015; Zheng *et al.* 2017). The runtime required by several widely used tools to analyze datasets of <10k cells can range from tens of seconds to several days, especially for those that predict the cell types at the single-cell level rather than at the cluster level (Abdelaal *et al.* 2019; Huang *et al.* 2021).

In this work, we present a new cell annotation system, CellAnn. The system includes more than 350 preprocessed reference datasets, including the major tissue types in human and mouse. Users can easily search the relevant reference datasets for comparison. Furthermore, a newly designed algorithm can produce the results with high accuracy and high speed. Our algorithm assigns the cell types to single-cell clusters, and therefore, the analysis can be done very fast. We assessed the performance of our algorithm and showed it is superior to existing methods. Finally, all the analyses are done on a user-friendly online web server, which is available at www.cellann.io.

## 2 Materials and methods

### 2.1 Data structure

**Figure 1.**
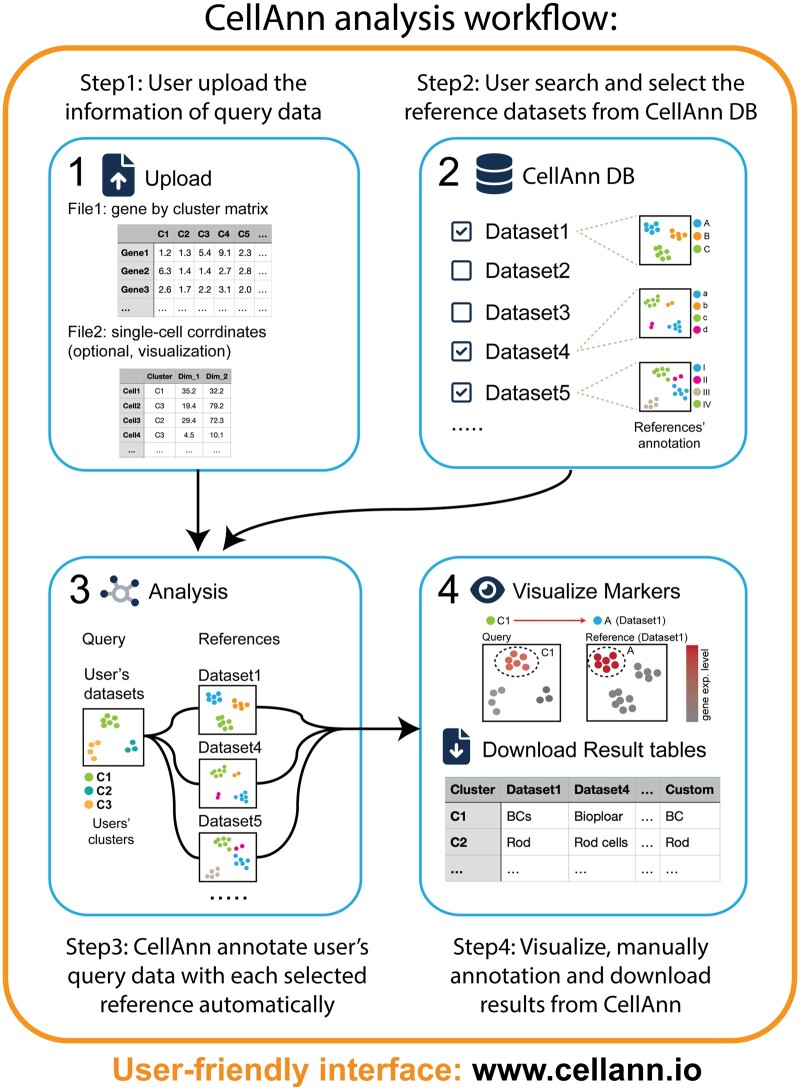
Schematic diagram of the CellAnn workflow. CellAnn has four major steps. (A) uploading the gene expression profile of the query clusters, (B) selecting references in the CellAnn database, (C) predicting cell types based on the selected references, and (D) checking marker gene expression patterns, harmonizing multiple predicted cell type labels, and downloading the final result.

The usage of CellAnn is straightforward. First, users can upload the average gene expression for each cluster (gene by cluster matrix) to the system (Fig. 1; Step 1). Then users can search the CellAnn database for one or multiple references within the selected tissue type(s), such as “retina” or “liver” (Step 2). The system will then compare the query dataset to the selected reference dataset(s), and the cell annotation results will be generated and made available for download (Step 3). If users wish to visualize the results, they may upload a single-cell coordinate file. The results of the cell annotation and marked gene expression patterns can then be viewed and examined (Step 4).

### 2.2 Source of CellAnn database

We downloaded the raw gene expression matrix and author-annotated cell type information from UCSC Cell Browser (cells.ucsc.edu), Single Cell Portal (singlecell.broadinstitute.org), CELLxGENE (cellxgene.cziscience.com), and GEO (Gene Expression Omnibus). We also collected single-cell atlas datasets from HCL (https://db.cngb.org/HCL/) and MCL (https://bis.zju.edu.cn/MCA/). We selected a diverse set of data that covered as many tissue types as possible. For a given tissue type, we preferred large datasets with many cell types. We did not include the datasets from diseased samples (e.g. cancers) in the current version.

### 2.3 CellAnn query dataset preparation

To calculate the average gene expression profiles for each user-defined cluster, we summed up the raw gene expression counts of all cells for each cluster, normalized the gene expression by total counts, multiplied by a scale factor of 1e5, and took the natural logarithm of the obtained values [log(*x* + 1)]. We also provide tutorials and source code on the GitHub page of CellAnn for both Seurat and Scanpy users (https://github.com/Pinlyu3/CellAnn_shinyapp).

### 2.4 CellAnn reference dataset preparation

All the references in the CellAnn database are processed with the following steps:

- Preprocessing: First, after downloading the expression matrix and annotation, we split some huge single-cell atlas (e.g. “HCL,” “Tabula Muris”) into several smaller datasets by tissue types. Next, we harmonized the gene names for each dataset. If the gene names are not the official gene symbol, we convert them to the GENCODE 42 (Human) and GENCODE M31 (Mouse) gene annotations. Then, we normalize the gene expression values of each cell by its total expression, multiply the result by a scale factor (1e5), and take the natural logarithm of the obtained values (log(*x*+1)).
- Preparing the reference-specific background reference dataset: We introduce the background reference because it can be used to calibrate the similarity between the query dataset and selected reference datasets. For each reference dataset, we integrate it with single-cell atlas datasets (i.e. background) to obtain more specific marker genes and comparable expression values across all tissues. For human references, we used the “HCL” or “Tabula Sapiens” datasets as a background, and for mouse references, we used the “MCL” or “Tabula Muris” datasets as a background.
  Since our analysis will be performed at the cluster level, we calculate the average gene expression level for the clusters in the background and the reference dataset. First, we used the “scVI” software (Lopez *et al.* 2018) from scvi-tools (Gayoso et al.) to remove the batch effect between the reference and background datasets at the single-cell level. After integration, we obtained a combined dimension space and a combined, corrected cell-by-gene matrix that included all the single cells from the given reference and the background.
  Next, we obtain new clusters with all cells from both background and a reference dataset on the combined “scVI” dimension space using “FindNeighbors” and “FindClusters” functions from Seurat. We then calculated the average expression matrix of these new clusters using the combined, corrected single-cell gene expression matrix. The average expression matrix of the new clusters is denoted as Eb, in which rows represent genes and columns represent combined clusters.
  Next, we calculate the corrected average expression values of all the cell types in the selected reference datasets based on the combined clusters. For some cell types in the reference, the cells might be distributed to different clusters after the new clustering. For each given cell type in the reference, we calculate the percentage of cells in the new clusters and obtain a matrix of percentage, Wb, in which rows represent background clusters and columns represent the cell types in reference. Then, we calculate the cross product of matrix Eb and Wb to obtain the corrected expression matrix, Er, in which rows are genes and columns are the cell types in reference.
  Finally, we used the “COSG” package to identify marker genes across all the background clusters (denoted as bg-Markers), which will be used to calculate the correlation coefficient of gene expression between query and reference-specific background.
- Preparing the refined references: In some studies, authors may label multiple adjacent clusters as a single major cell type. Therefore, using the average expression of these major cell types may ignore the variance of the sub-cell types and confuse downstream analyses. To improve the sensitivity of our analysis, we first split the entire reference dataset by major cell types with the function “SplitObject.” Then, we re-performed clustering analysis for each split dataset using author-provided cell similarity graphs such as “UMAP” or “t-SNE.” If the authors do not provide the coordinate information, we calculate the UMAPs by the standard Seurat workflow. Next, we use the “FindNeighbors” and “FindClusters” functions with a resolution parameter of 0.3 to obtain refined clusters and label them as sub-cell types. Next, we get the average expression matrix (denoted as Ersub) by summing the raw counts in each sub-cluster and normalizing the expression value as described before. Finally, we calculate marker genes for original clusters (denoted as main-Markers) and sub-clusters (denoted as sub-Markers) using the “COSG” package.

### 2.5 Harmonize gene names between species

CellAnn enables users to perform cell type prediction across species. After uploading a gene expression matrix, CellAnn employs a predictive algorithm to determine the species from which the query dataset originates. This is accomplished by computing the overlap ratio of gene symbols between the input matrix and human or mouse gene annotations obtained from GENCODE 42 and GENCODE M31, respectively. If the ratio of overlapping genes exceeds 50% for either human or mouse gene symbols, CellAnn will label the query data with the corresponding species. If fewer than 50% of genes overlap, CellAnn will issue a “Warning” message, prompting the user to review their input files. In cases where the query data and reference datasets are from different species, CellAnn automatically converts gene names in the query data to enable compatibility with the reference datasets. The orthologous genes file is downloaded from the database (http://www.informatics.jax.org).

### 2.6 Comparison algorithm of CellAnn

**Figure 2.**
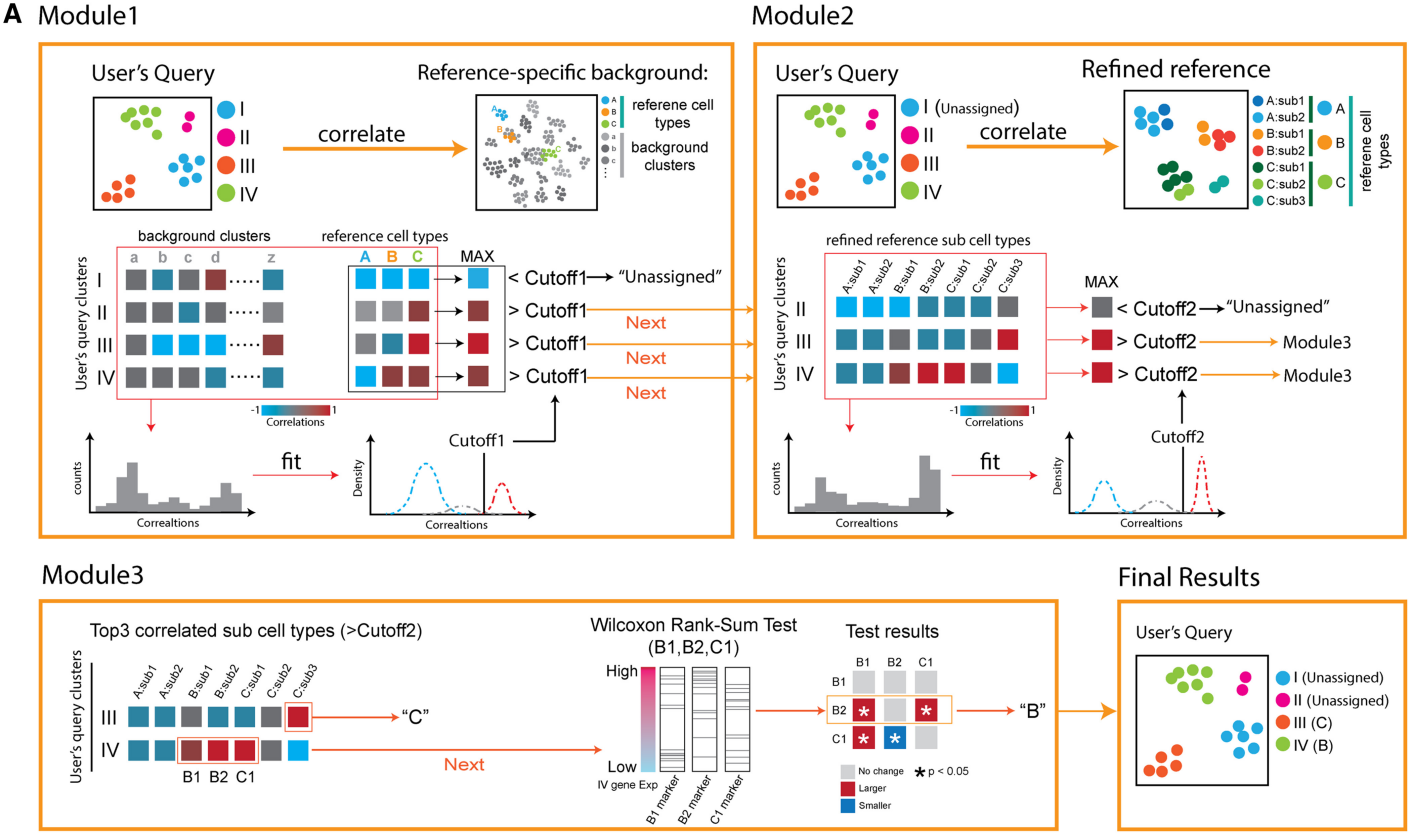
The algorithm design of CellAnn. (A) The CellAnn algorithm consists of three modules: (1) Module 1: CellAnn calculates the correlations between query clusters and clusters in reference and background. The cutoff is estimated based on the correlation between query clusters and background references. (2) Module 2: CellAnn calculates the correlations and estimates correlation cutoffs between the query data and sub-clusters in reference datasets. (3) Module 3: CellAnn performs the Wilcoxon rank-sum test to determine cell types further if a query cluster is similar to multiple sub-clusters in the reference.

We develop an algorithm to compare a query dataset and a selected reference dataset. The algorithm consists of 3 modules (Fig. 2A).

Module 1. We first determine a cutoff from the comparison of background and query datasets. To do so, we compute correlations between the query clusters (Eq) and background reference datasets (Eb) with the expression value of bg-Markers. We obtain nb *nq coefficient values, where nb and nq equal the number of clusters in the background and query, respectively. We then obtain a distribution of the nb *nq correlation coefficients. Since the background reference contains a diverse set of tissue types, most query cluster-background cluster pairs are from different cell types. We assume that the observed distribution composes a negative distribution (from pairs with different cell types) and a positive distribution (from pairs with the same cell types).

Based on the assumption, we decompose the density distribution into one to three Gaussian distributions using the mclust package in R. If the observed distribution is better fitted by only one Gaussian distribution, the distribution is considered from different cell types. We then set the correlation cutoff as the point whose cumulative probability exceeds 0.75 in the distribution. If two or three Gaussian distributions can better explain the observed distribution, the distribution with the largest μ is considered from the same cell types, and the remaining distributions are considered from different cell types. For two Gaussian distributions, we set the intersection point as cutoff. For three Gaussian distributions, we set the cutoff as the point whose cumulative probability exceeds 0.75 in the second distribution. Finally, to avoid the extreme cutoff values, we set the minimal and maximal cutoff values to 0.4 and 0.6, respectively, if the obtained cutoff falls out the range of 0.4–0.6.

We then calculate the correlations between the query clusters (Eq) and reference datasets (Er) according to the expression value of bg-Markers. For a given query cluster, if all its correlations with reference cell types are lower than the cutoff, we label it as an “unassigned” cluster. Otherwise, we go to next Module.

Module 2. We next calculate the pairwise correlation coefficients between the query clusters (Eq) and the sub-clusters (Er-sub) in the reference (Fig. 2A, step 2) according to the expression value of main-Markers. We then obtain a distribution of the coefficients. Similar to the above module, we fit the distribution with one to three Gaussian distributions and determine a cutoff for this specific query dataset. For each query cluster, if the maximal coefficient with the sub-clusters in the selected reference is below the cutoff, the query cluster will be assigned as “unassigned.” If only one correlation coefficient for a given query cluster is above the cutoff, the query cluster is assigned to the cell type associated with the sub-cluster in the reference. If more than one coefficient is above the cutoff, especially if the matched sub-clusters belong to different cell types in the original annotation, we go to next Module.

Module 3. We perform additional statistical tests to determine the cell type of the query cluster. First, we select the top three sub-clusters whose correlations are larger than the cutoff. Next, we extract the marker genes of the top three sub-clusters from the sub-Markers list. Then, we use the Wilcoxon Rank-Sum test to make pairwise comparisons among these three groups of marker genes to check whether their expression values are significantly higher than the others in the query cluster. Based on the statistical results, we assign the cell type of the sub-cluster with the highest significance as the predicted cell type of the given query cluster.

### 2.7 Evaluation of computational scalability

To measure the computational resources required for running CellAnn and other comparable methods, we simulate a query and several reference samples with different sizes from the PBMC datasets. We first downloaded PBMC data from the single-cell portal (SCP424). The original datasets contain a total of 31 021 cells from 8 different sequencing libraries. We use the “inDrops” PBMC datasets as the query datasets. For reference datasets, we perform downsampling from entire dataset with sizes of 1k, 5k, 10k, 15k, 20k, and 25k.

We use the function “system.time()” in R to evaluate the running time of all the methods. In addition, we use the R package “Bench” to measure the maximum memory usage when running all the software. All the efficiency tests were performed on a Linux server with Intel(R) Xeon(R) Gold 6126 CPU processors and 790 GB physical memory.

### 2.8 Comparison with other cell annotation methods

We compare CellAnn with five competing methods, including scmap, CHETAH, scClassify, scPred, and Seurat v4. To compare the methods, we have prepared a total of 52 benchmark tests using the “Tabula Muris” single-cell atlas dataset. The “Tabula Muris” single-cell atlas contains single-cell data from different tissues that were sequenced using two different platforms: fluorescence-activated cell sorting (FACS) and droplet-based sequencing (droplet). We often use datasets from one platform as a reference and predict the cell types in datasets generated from another platform.

We prepared both cell- and cluster-based inputs for these competing methods as follows. For CHETAH, scClassify, scPred, and Seurat v4, both the query and reference datasets are single-cell datasets. We use the log-normalized raw-counts matrix as the cell-based expression profiles for both query and reference datasets in these methods. For scmap, the query datasets are cluster-based expression profiles, while the reference datasets are single-cell-based expression profiles. To prepare the cluster-based inputs, we re-performed clustering analysis with Seurat for each cell type in the query dataset and calculated the average expression profile of all the new clusters. For CellAnn, the query and reference datasets are both cluster-based expression profiles. The background of the query dataset is “Tabula Muris” datasets.

We used the following parameters for these methods. In scmap-cluster, we set the similarity threshold to 0.5, and cells with a predicted score below this threshold were labeled as “unassigned.” In Seurat V4, default settings were used to find anchors between the query and reference datasets. The number of principal components was set to 50, and cells with a predicted score < 0.5 were also categorized as “unassigned.” For scClassify, we constructed the hierarchical tree using the “HC” method and classified the ensemble model using the “WKNN” method. The “limma” method was used to select genes, and both the “Pearson” and “cosine” methods were employed to measure the similarity between single cells. In CHETAH, we used all default parameters to train the model and make predictions for the query datasets. In scPred, we trained the reference datasets using the “svmRadial” model and set the threshold for probabilities to classify cells into groups at 0.55; cells below this threshold were labeled as “unassigned.” Finally, in CellAnn, we did not adjust any parameters during comparison, and default settings were used.

## 3 Results

### 3.1 Reference dataset collection

**Figure 3.**
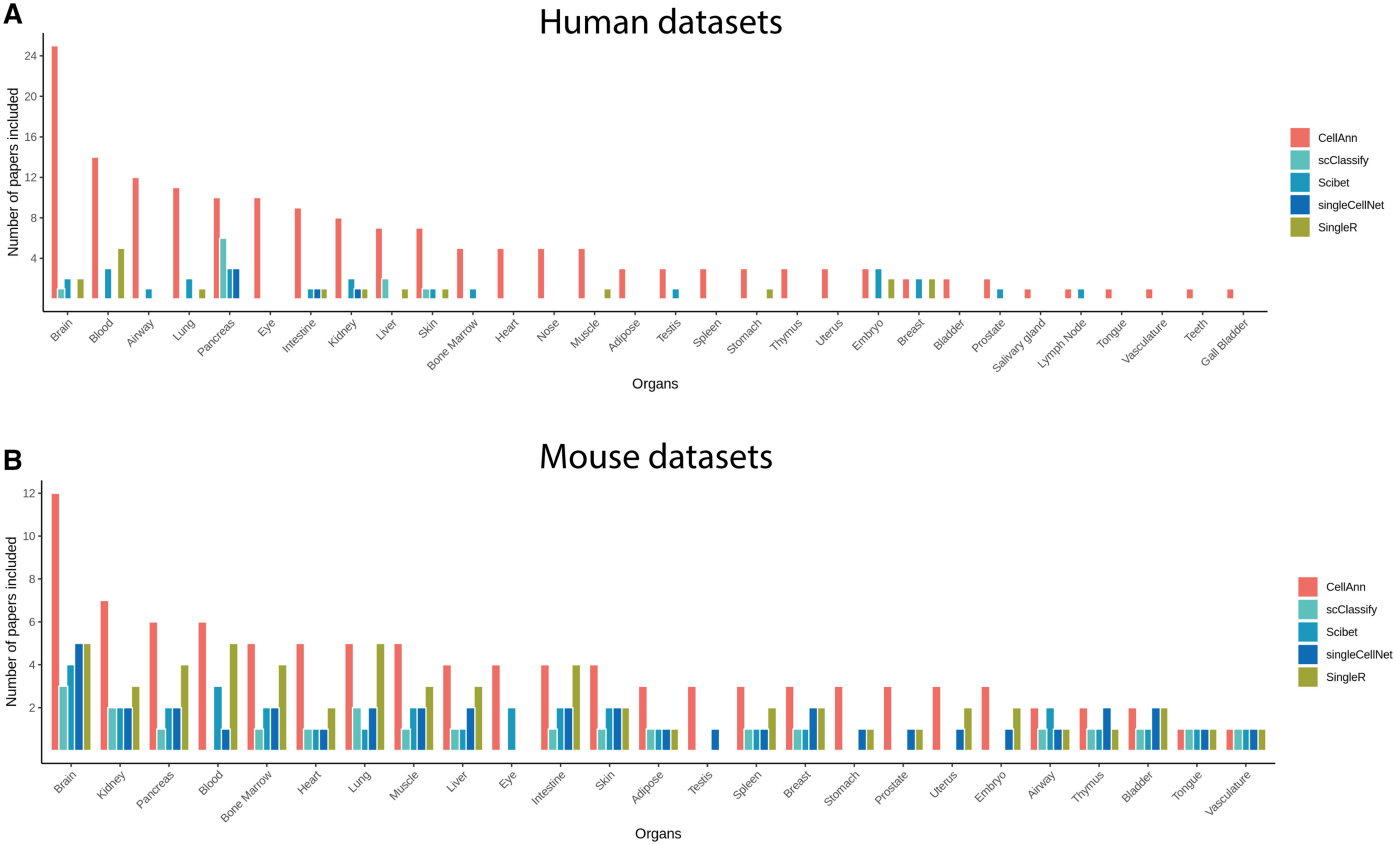
Reference datasets included in CellAnn. The bar plot compares the available datasets for each organ type in human (A) and mouse (B) included in the CellAnn database and other reference-based cell annotation web services. The *x*-axis represents different organs, and the *y*-axis represents the number of papers related to the corresponding organs.

Cellann database contains 204 and 191 nonredundant scRNA-seq datasets for humans and mice, respectively. The number of associated studies (papers) of these datasets in CellAnn is significantly larger than other reference-based annotation tools (Fig. 3A and B). For example, CellAnn contains single datasets from more than 109 (36) papers in Human (Mouse). However, scClassify, Scibet, singleCellNet, and singleR, four representative reference-based cell annotation databases, include only 11(5), 31(17), 6(6), and 19(26) papers, respectively (Fig. 3A).

The reference datasets cover most of the major tissue types in the two species. Some tissue types are well-studied, such as the brain, blood, airway, and lung. Therefore, more datasets were obtained from these tissues. Most of the collected tissue types have more than one single-cell datasets, allowing for multiple-reference comparison with the query dataset.

### 3.2 Algorithm performance

**Figure 4.**
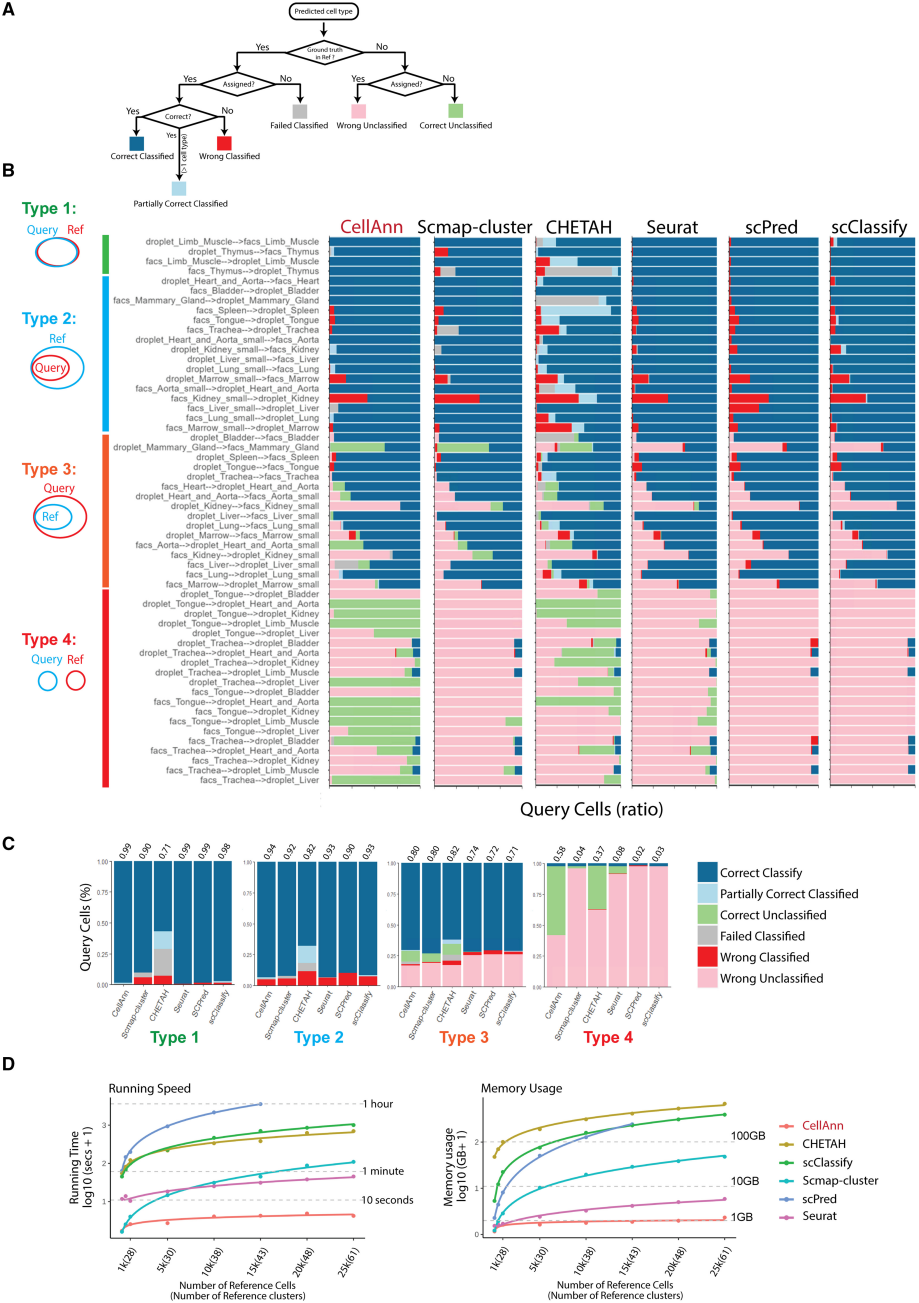
The performance and scalability of CellAnn. (A) Evaluation framework of CellAnn. According to the author’s annotation, predictions are classified into “Correct Classified,” “Correct Classified (Partially),” “Wrong Classified,” “Failed Classified,” “Wrong Unclassified,” or “Correct Unclassified.” (B) The benchmark results of 56 testing pairs for 6 different methods. Each bar indicates the composition of predicted cell types. Based on the overlapping of cell types between query and reference datasets, we divided these test pairs into four groups: type 1, type 2, type 3, and type 4. The Venn diagrams on the left show the relationships of type 1–type 4. (C) The bar plots indicate the composition of predicted categories of the average performance in a collection of reference–testing pairs. (D) Benchmarking the efficiency of CellAnn. Left: the line plot shows the running time under the default settings of each algorithm. Right: the line plot shows the peak memory usage of each algorithm. The *x*-axis is labeled by the number of cells and the clusters in the references. The curves are truncated if a method is not scalable to a certain size of the references.

We first evaluate the performance of our comparison algorithm (Fig. 4). Using annotation in the reference dataset as ground truth, we have six outcomes: correctly classified, partially correctly classified, correctly unclassified, failed classified, wrongly classified, and wrongly unclassified (Fig. 4A). The first three are considered correct predictions, while the latter three are wrong predictions.

To comprehensively assess the performance of our method, we tested four types of situations (Fig. 4B). In Type 1, the cell types in the query and reference datasets are exactly the same but from different platforms. In Type 2, the cell types in the query are the subset of the cell types in the reference. For the first two types, we expect, in the ideal situation, to find all the cell types for the query clusters. In Type 3, the cell types in the reference are the subset of the cell types in the query. Some query clusters are not expected to have an assignment (i.e. unclassified). In Type 4, the cell types in the query and reference are not overlapped. All the query clusters should be unassigned. The performance in Types 3 and 4 comparison is critical for a database search because the users might select remotely similar or even irrelevant datasets as references.

We compared our algorithm with popular cell annotation methods, including Scmap-cluster (Kiselev *et al.* 2018), CHEATAH (de Kanter *et al.* 2019), Seurat (Hao *et al.* 2021), SCPred (Alquicira-Hernandez *et al.* 2019), and scClassify (Lin *et al.* 2020) (Fig. 4B and C). The performance of our algorithm in Type I comparison is comparable with Seurat, SCPred, and scClassify. They all achieved ∼0.99 correct predictions. However, Scmap-cluster and CHEATAH have lower success rates of 0.90 and 0.71, respectively. In Type 2, CellAnn reached a success rate of 0.94, outperforming other methods. Furthermore, for types 3 and 4, CellAnn still has a good success rate. However, CHEATAH, which has worse performance in the types 1 and 2 comparisons, has a relatively good success rate in types 3 and 4. This might be due to a more stringent cutoff selection for CHEATAH. In contrast, CellAnn uses an automatic query-specific cutoff selection, which might be the reason for a high success rate in all types of comparisons.

### 3.3 Speed and memory use

We then assess the speed and memory used for CellAnn and other existing methods. We use the reference datasets with different cell numbers and different numbers of clusters, ranging from 1k to 25k cells and from 28 to 61 cell clusters (Fig. 4D). CellAnn is the fastest and finishes the jobs within 10 s, making it suitable for an online cell annotation tool. Seurat and Scmap-cluster are also fast, finishing the jobs within minutes. The other three methods, CHETAH, scClassify, and scPred are suitable small reference datasets.

Similarly, CellAnn is also efficient in memory use because it is a cluster-based, rather than a cell-based approach (Fig. 4D). The peak memory required for Cell Ann is around 1 GB with the largest dataset (25k cells). Seurat requires around 5 GB for the largest reference dataset. The memory needed for other methods easily exceeds 10 GB if the reference datasets contain more than 25k cells.

### 3.4 Case study

**Figure 5.**
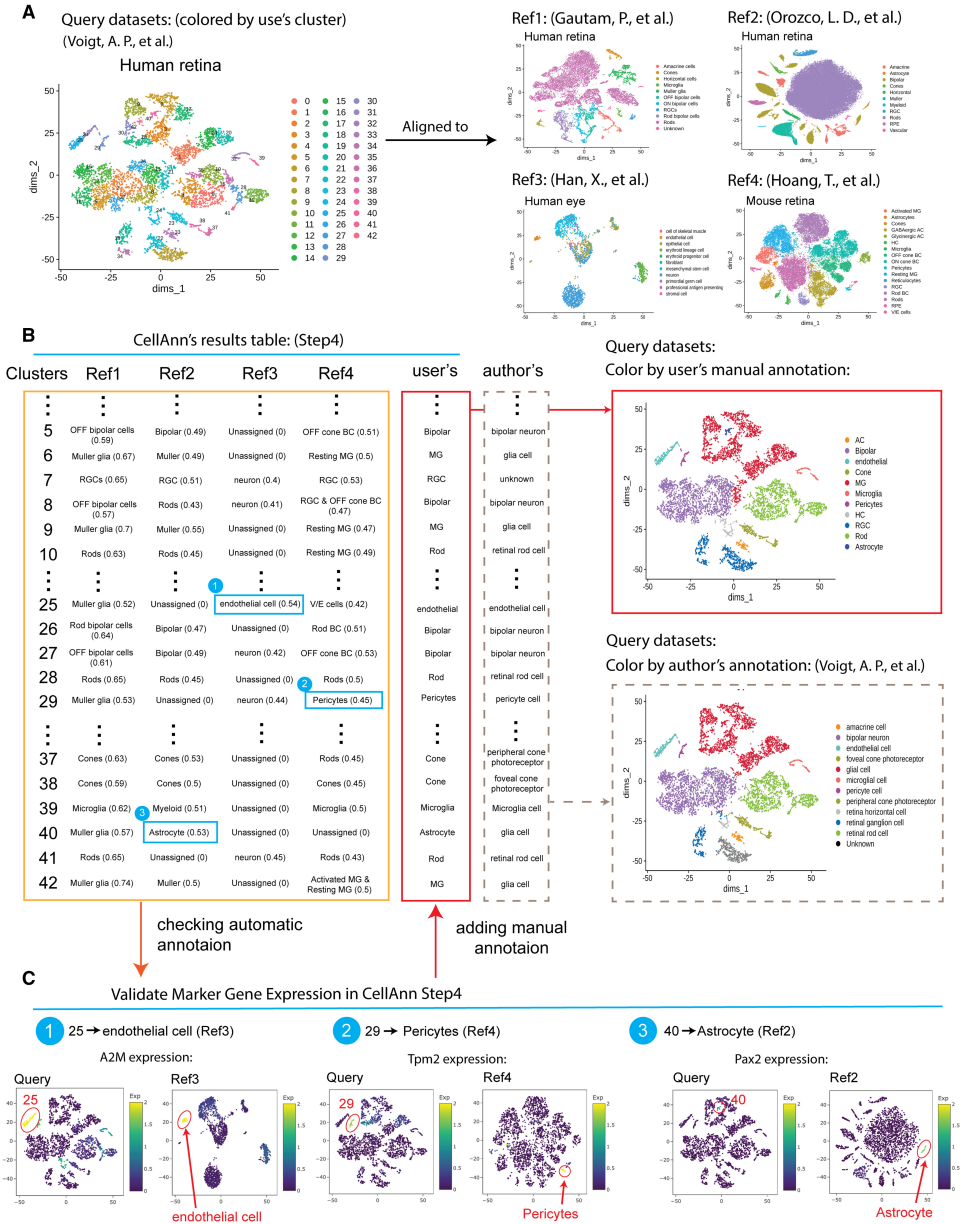
A case study on cell type annotation with multiple references using CellAnn. (A) The UMAP plots show the query data and four different reference datasets. The cells in the query data are colored by clusters, and the cells in the reference datasets are colored based on the author's cell type annotation. (B) The comparisons of alignment results by CellAnn with the author's annotation in the query datasets. The left panel displays a table that shows the predicted cell type labels by CellAnn. The right panel displays UMAP plots of the query datasets, with the predicted annotation and the author's original annotation. (C) The marker gene expression levels in both query and reference datasets, help the users to select the cell type when the results from multiple reference datasets are inconsistent.

Multiple reference datasets can provide a more confident annotation. Here we use an example to demonstrate its usage. We used one published human retina dataset as input (Voigt *et al.* 2019), which included 43 clusters (Fig. 5A). We then selected four relevant datasets as references (Han *et al.* 2020; Hoang *et al.* 2020; Orozco *et al.* 2020; Gautam *et al.* 2021) (Fig. 5B). The references have three datasets from the human retina or eye and one dataset from mouse retina. Using default parameters, we obtained the predicted cell annotation for the 43 input clusters (Fig. 5C). The cell type labels from the four datasets could be different. For example, both “bipolar” and “BP” refer to the same cell type, while “Muller glia,” “Muller,” and “Resting MG” are the Muller glia cell type. Overall, the annotation from different references is consistent, increasing our confidence level in the cell annotation. However, some clusters have inconsistent annotations (see highlighted blue boxes in Fig. 5C). The top marker genes of the clusters obtained from the reference can be used to choose the winners of the annotations for the input clusters. The second to the last column is our summary annotation after integrating the prediction from the four references, while the last column shows the original annotation from the published input dataset. The agreement between the prediction and the original annotation suggested the high quality of the predicted annotation.

### 3.5 User interface

**Figure 6.**
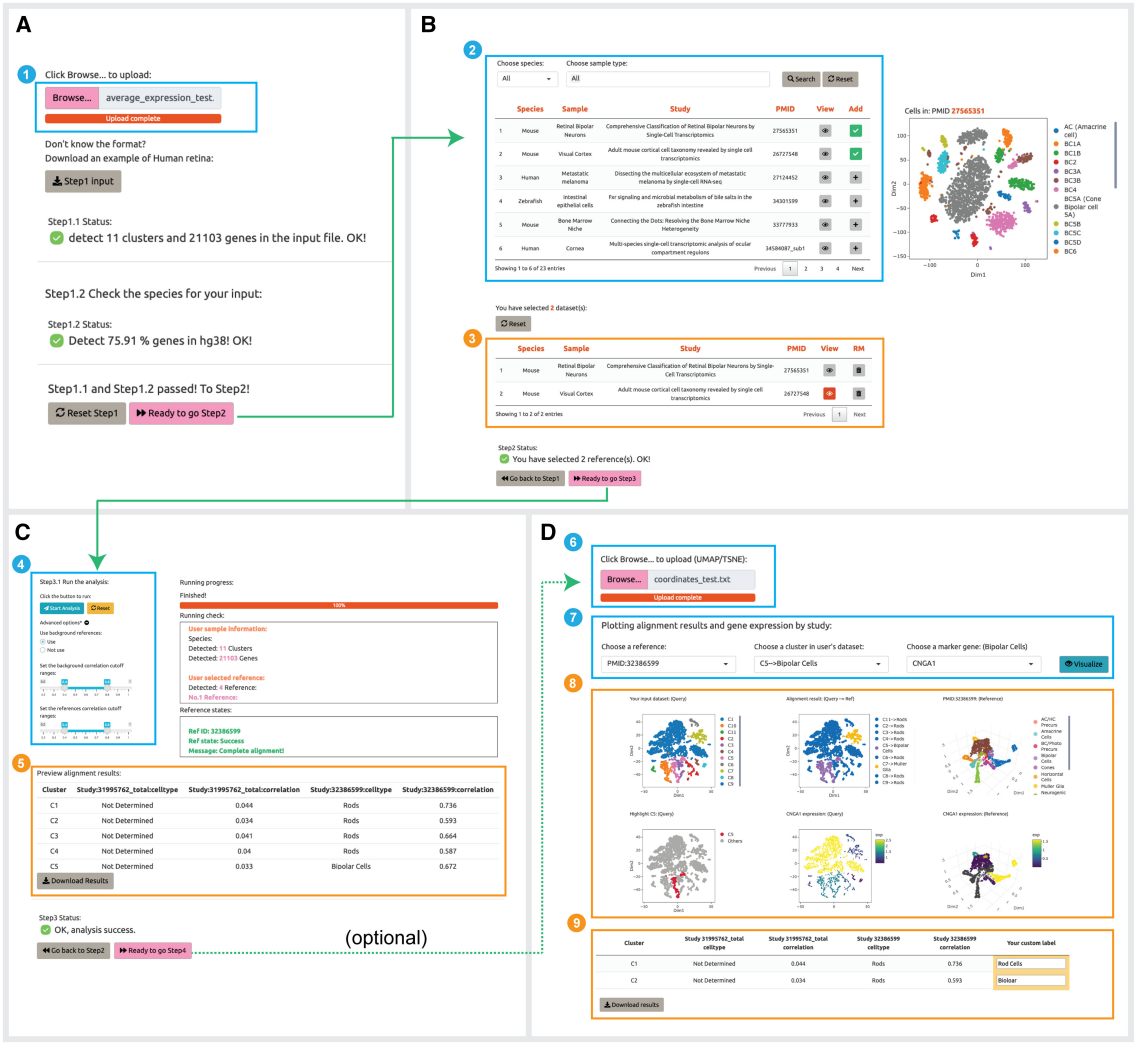
Example webpages associated with each step of the analysis. (A) Step 1: a window for uploading expression profiles of query data (1). (B) Step 2: a table for searching and selecting datasets in CellAnn (2), and a table for reviewing the selected datasets (3). (C) Step 3: a window for running CellAnn and tuning parameters (4). a table for downloading predicted cell types (5). (D) Step 4: a window for uploading coordinate profiles (6), a search window for alignment results and related marker genes (7), UMAPs for query and reference data that are colored by cluster, predicted cell type, author's annotation (for reference), and gene expression values (8), and a downloadable and editable table that users can review to see predictions and add their own custom annotations (9).

We have designed an interactive web server for CellAnn (www.cellann.io). To use the server, users first perform clustering analysis using other methods (e.g. Seurat), and calculate the average expression profiles for the clusters. Users can then upload the cluster versus gene matrix to the server (Fig. 6). The server will automatically check the number of clusters, genes, and species based on the user's input file. If the user's input is correct, users will be able to proceed to the next step. Otherwise, a warning message will be shown.

Users are able to select one or more relevant datasets as references. The current version of CellAnn contains 204 human and 191 mouse single-cell datasets, respectively. To facilitate the identification of relevant datasets, we have organized the datasets into 32 organs. Users can search for the relevant datasets by organ. We also provide a preview button for each dataset, allowing users to preview the dimension plot for each study and check which cell types are included in the reference dataset.

The users can then run the analysis. While the whole analysis procedure is automatic, users also have the option to set the range of cutoff values in the user interface. The default range for the cutoff value is 0.4 to 0.6. The analysis results will be presented in a table format.

The final step is optional. If users upload an additional file with the cell clustering coordinates, we will provide the visualization of the annotation analysis. As an independent validation, users can also select marker genes for a particular cluster from reference clusters and examine the expression level of the genes in query clusters.

CellAnn is a freely accessible web server available at www.cellann.io. The help page of the web server provides brief documentation. CellAnn is compatible with all commonly used web browsers, including Safari, Chrome, Opera, Firefox, and Microsoft Edge. The underlying data of CellAnn is also freely accessible on the GitHub page of the web server.

## 4 Discussion

We develop a method, CellAnn, for single-cell annotation. The method has the following unique advantages. First, it is a cluster-based algorithm. Compared to cell-based methods, it is very fast. This is important, especially if we want to compare multiple reference datasets. Second, it contains many preprocessed single-cell datasets as references. The large collection of references makes it convenient to use and saves a lot of time for the users. Third, the performance of our algorithm is robust. One reason for the robustness is that we introduce a background reference and use it to calibrate the choice of the cutoff.

One interesting question is whether the cell types are discrete or continuous. A cell type will undergo transcriptomic changes under certain conditions, such as disease and aging. Should we consider them as the same cell types or annotate them as distinct diseased- or aged-cell types? In the current version of CellAnn, we did not include the datasets with diseases (e.g. cancers). We plan to include more datasets in the next version so that the users will obtain not only the predicted cell types but also the associated conditions.

If users selected multiple reference datasets, we presented the predicted cell types from each reference dataset and did not provide a consensus score or an “averaged” result from the multiple reference datasets. There are several practical issues for this approach. First, we do not want to adopt a voting strategy to provide an averaged result because the quality of the datasets is not the same. Second, the reference datasets might use different cell type nomenclatures and make it difficult to “average” the results. For example, “Muller glia,” “Muller,” and “MG” are the same cell type, and “photoreceptor,” “rods,” and “cones” could be the same cell type, too. To make things more complicated, “MG-1,” “MG-resting,” and “MG-activated” could be the Muller glia at different conditions. Therefore, we decided to keep the original annotation from the publications and not try to standardize the cell-type annotation.

One potential problem for the reference-based methods is that the methods rely on the quality of reference datasets and the query dataset. The low quality of the query dataset or incorrect annotation in the reference datasets will cause false predictions. We will keep updating the database when new datasets become available and believe that adding new datasets will make CellAnn even more powerful.
